# Evidence-based telemedicine for veterinarian care of livestock

**DOI:** 10.3389/fvets.2026.1818287

**Published:** 2026-06-25

**Authors:** Mark A. M. Kramer, Peter M. Roth

**Affiliations:** 1 Computational Medicine, University of Veterinary Medicine, Vienna, Austria; 2 Computer Science Department, Webster Vienna Private University, Vienna, Austria

**Keywords:** Austria, PLF, precision livestock farming, sensors, telemedicine, veterinary care

## Abstract

Our research aims to demonstrate that veterinary care can be assessed and managed digitally through continuous monitoring, thus making the veterinary profession in rural areas more attractive and sustainable. The prototype of a telemedicine and teletriage architecture was developed using proven technology platforms (standardized IT services, secure cloud storage, and existing communication protocols). The ultimate objective is to create a model that demonstrates that digital tools can legally and effectively support rural veterinarians. The implementation reveals a distinct gap in the readiness levels of its two core components: the telecommunications backbone and the biometric sensor network. We deploy an experimental sensor network prototype to validate its ability to collect and interpret physiological and behavioral data for early disease detection, providing a proof of concept that the evidence-based veterinary telemedicine infrastructure is technically viable; however, the integrated sensor network requires further clinical validation in live animals to confirm its diagnostic effectiveness.

## 1 Introduction

The livestock farming sector is a crucial part of the country's infrastructure, vital to maintaining a reliable food supply. However, the resilience of this sector is challenged by significant challenges, including feeding a growing population and addressing severe environmental crises such as depletion of natural resources and climate change. The health of livestock is dependent not only on the farmers who care for them daily but also on access to skilled veterinarians. Thus, it is important to make better use of the available resources in veterinary care, particularly important given the increasing shortage of food animal practitioners which can be observed worldwide (1–8). The reasons are manifold: On the one hand, there is a demographic shift whereby an increasing number of students from urban areas are entering veterinary medicine, yet lack a connection to livestock farming in particular and to agriculture in general (9, 10). On the other hand, aspects such as general job satisfaction, work-life balance, time for family, and quality of life are becoming increasingly important (3, 11–13).

Currently, livestock veterinary medicine operates on a curative (reactive) model: intervention occurs only after an acute illness is detected. This approach can be considered resource-intensive and inefficient. Although preventive approaches exist, they are not yet the standard. Thus, we adopt a holistic approach to ensuring sustainable livestock to address this specific challenge of the current system. By integrating digital technologies, we aim to establish a scientific foundation for a system that covers both curative and preventive aspects. Furthermore, we address the urgent need to attract the next generation of veterinarians to rural practice.

The sector must adapt to the expectations of younger veterinarians who prioritize work-life balance over the traditional 24/7 on-call model.

Thus, we propose to pursue alternative strategies in order to address these challenges. In particular, the use of digital technologies and the availability of a broader data base should also make the profession of food animal practitioner more attractive (11). On the one hand, because even in emergencies, necessary information can be accessed in real-time before and during interventions on the animal. On the other hand, because digital technologies enable better collaboration and communication between the parties involved. This allows treatments and interventions to be better planned, thereby reducing travel distances and the associated time expenditure—for example through a reduction in the number of unnecessary call-outs and improved route planning.

## 2 Methods

The core approach focuses on a holistic strategy that integrates curative veterinary medicine with preventive herd monitoring to facilitate data-driven decision-making. Using digital technologies, we aim to establish a system that enables veterinarians to access real-time data to support teletriage, allowing them to assess emergencies before arrival and optimize intervention planning. This evidence-based approach would employ continuous 24-hour monitoring of livestock to detect health issues early, shifting the focus from purely reactive treatments to proactive health management, thus improving animal welfare and improving veterinary efficiency by better utilizing resources.

To realize this strategy, we created two critical technical components: a robust communications infrastructure for telemedicine and teletriage and a sensor-based monitoring network to support continuous health monitoring. Our working prototype focuses on developing the technical backbone needed to ensure seamless data exchange and communication between agricultural operations and veterinary service providers, laying the foundation for practical connected care. Currently, we deploy an experimental sensor network prototype which integrates diverse data sources–such as accelerometers, cameras, and automated feeding systems–to validate its ability to collect and interpret physiological and behavioral data for early disease detection.

To facilitate rapid development, we created a digital twin of the functional hardware of the prototype. This prototype-mirror setup enables continuous testing and iteration of sensor integration and data transmission protocols in a local environment, eliminating the need to travel to the primary research site for frequent physical visits during the initial development phases. In addition, by validating existing telemedicine solutions and remote assessment tools, our research demonstrates how digitalization can reduce the physical burden of unnecessary emergency drives. This shift toward a data-driven, flexible workflow transforms the rural veterinarian's role from a “firefighter” to a remote “herd manager”, making the profession significantly more attractive to a tech-savvy demographic that might otherwise migrate to small-animal medicine.

### 2.1 The “Living Lab” environment

**Figure 1:**
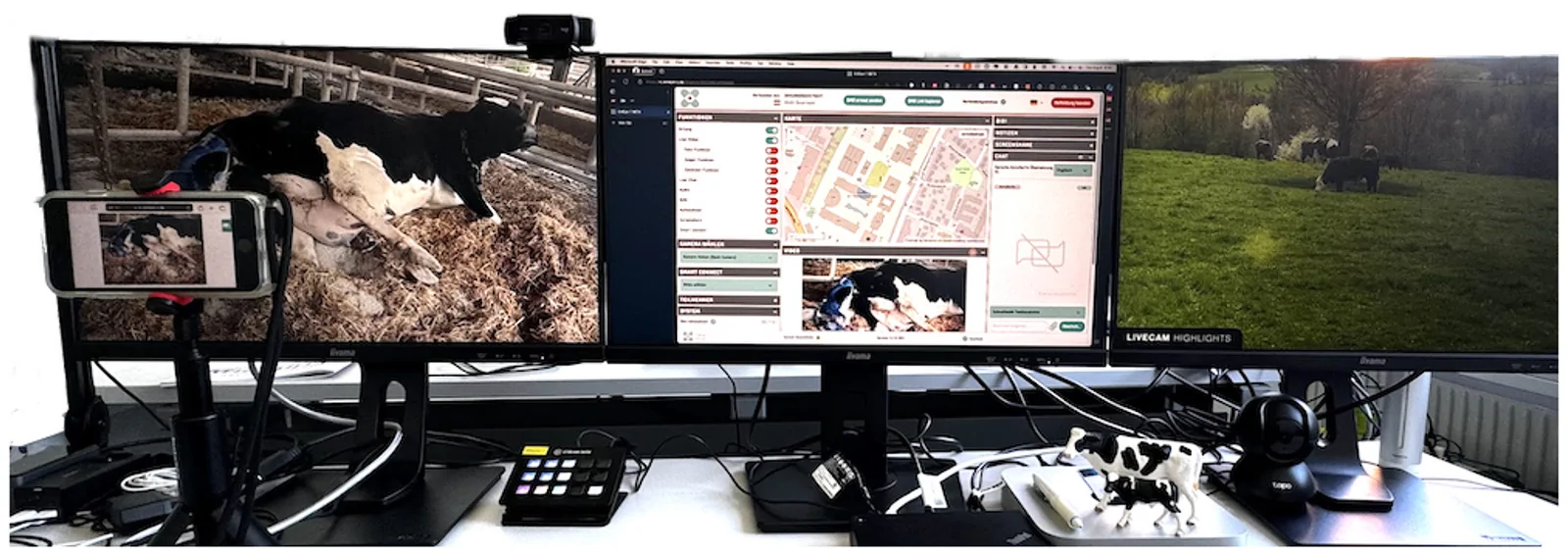
Telemedicine/tele-triage setup.

**Figure 2:**
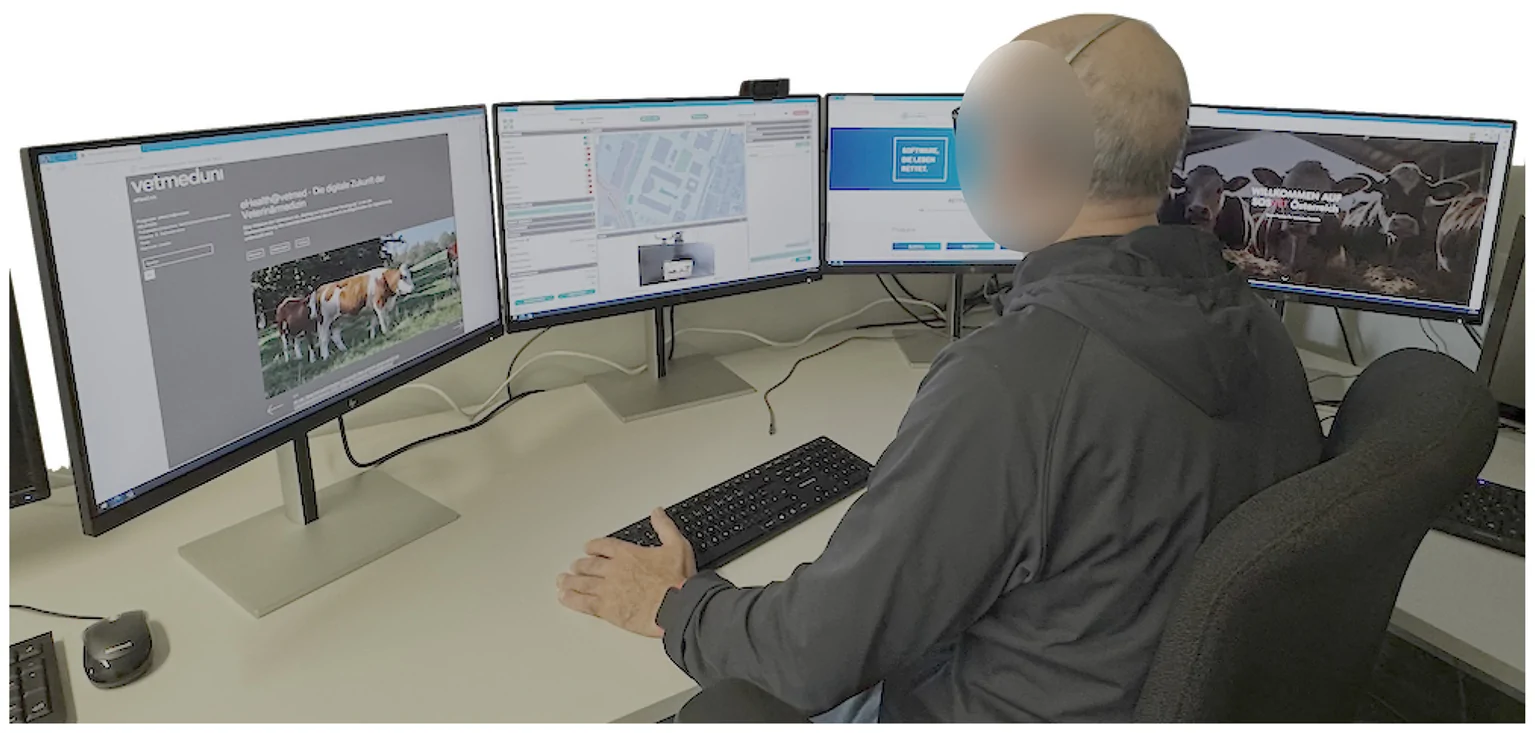
Testing EmergencyEye with a simulated telemedicine event.

To enable remote veterinary decision-making, we developed a specialized IT infrastructure capable of handling high-volume data streams. To process incoming data effectively for tele-triage, the system employs high-performance computing (HPC) resources. For the project's tele-triage interface (see Figure 1), we selected the EmergencyEye^®^ platform (14) due to its highly practical, browser-based architecture that requires no prior app installation (see Figure 2).

### 2.2 Prototyping the sensor network

In parallel with establishing the IT backbone, we developed a sensor network prototype to generate the “medical evidence” needed for accurate remote diagnosis. This methodological approach involved a comprehensive suite of monitoring technologies, including a network of IP cameras to capture behavioral data and a biometric array integrating accelerometers, temperature sensors, and RFID tags to track individual animal activity.

The primary methodological goal of this infrastructure is to use Computer Vision (CV) and Machine Learning (ML) algorithms to analyze collected sensor data, enabling the automatic detection of anomalies such as lameness or metabolic disorders.

To ensure replicability of the proposed architecture, the technical components were divided into a functional telecommunications backbone and an experimental sensor network (see Table 1). The digital twin allowed for continuous iteration of these hardware prototypes, ensuring that data transmission protocols were rigorously tested before full-scale deployment in the research facility.

**Table 1:** Technical specifications of the telemedicine and sensor infrastructure.

Component	Technical specification/platform	Purpose in study
IT backbone	High-performance workstations	Secure communication layer and high-performance data processing.
Tele-triage interface	EmergencyEye^®^ (browser-based)	Real-time video consultation and remote assessment without app installation.
Prototyping hardware	Raspberry Pi single-board computers	Agile testing of sensor integration and data transmission protocols.
Visual monitoring	Network of IP cameras & Computer Vision (CV)	Capture and analysis of animal behavioral data (e.g., lameness detection).
Biometric array	Accelerometers, Temperature sensors, RFID tags	Collection of physiological parameters for early disease detection.

To effectively validate the system, the solution was prototyped using energy-efficient single-board computers, specifically Raspberry Pi models. This approach allowed us to develop and test several “smart farm” physical sensor-based prototypes alongside a local network of IP cameras. This agile prototyping method ensured that the technical architecture for continuous data collection and transmission could be rigorously tested and refined prior to full-scale implementation, supporting the project's goal of integrating preventive monitoring with curative veterinary intervention.

## 3 Results

The implementation of our research prototype reveals a distinct bifurcation in the readiness levels of its two core components: the telecommunication backbone and the biometric sensor network. Although the digital infrastructure for remote consultation is fully operational, the hardware for automated animal monitoring remains in a developmental stage.

### 3.1 Telemedicine infrastructure: fully functional

The results indicate that the telemedicine and tele-triage backbone is fully established and functional. Using proven technology platforms–including standardized IT services, secure cloud storage, and existing communication protocols–we have successfully created a stable environment for digital veterinary interaction.

- Operational Capacity: The computational capacity to handle complex data analysis is operational, with servers successfully deployed.
- System Integration: This confirms that the technological barrier to remote consultation–connecting a veterinarian to a farm digitally–has been effectively addressed using standard, securely integrated enterprise solutions.
- Interface Validation: Testing with the EmergencyEye® platform demonstrated a reliable, browser-based video-communication system that requires no prior app installation, matching the standards used by emergency coordination centers.

### 3.2 Sensor network: a functional prototype

In contrast to the IT backbone, the sensor network remains a functional prototype. Although the prototype is equipped with the necessary hardware and sensors to detect parameters and is currently streaming data within the research environment, the full integration of these sensors into a closed-loop diagnostic system is ongoing.

- Data Transmission: The hardware architecture has been successfully integrated and tested for data transmission stability within the research environment.
- Validation Status: The system has not yet been deployed in live livestock for clinical validation. Consequently, the ability of sensor data to automatically trigger validated veterinary alerts remains under evaluation.
- Scalability: While the system is technically capable of continuous data collection, it has not yet been deployed as a standalone diagnostic tool for commercial livestock outside controlled research settings.

These results underscore the readiness of the communication infrastructure while highlighting the specific technical and clinical challenges that remain for the sensor network. The following discussion explores how these differing levels of technological maturity affect the practical implementation of Evidence-Based Telemedicine and outlines the necessary steps to move from a research prototype to a validated diagnostic system.

## 4 Discussion

To bridge the gap between our functional prototype and widespread adoption, we propose establishing regional “Living laboratories” to enable veterinarians and farmers to test telemedicine/tele-triage tools. The successful deployment of the prototype provides a proof of concept that evidence-based telemedicine is technically viable and location-independent. The technology works: the next step is contextual adaptation. We recommend establishing additional decentralized living laboratories where farmers and veterinarians can pilot these data-driven tools in a legally secure “sandbox” environment. This approach allows stakeholders to test and iterate the system without legal risk, ensuring that the final product meets the practical requirements of both livestock producers and veterinary practitioners.

### 4.1 Current state and future directions

Although this study demonstrates the technical feasibility of an integrated telemedicine and teletriage infrastructure, there is a long way to go. First, the current results reflect a validated IT architecture awaiting integration with live biological data streams. Although the hardware architecture–including accelerometers, and camera systems–has been successfully tested for stable data transmission (see Table 1, *Technical Specifications of the Telemedicine and Sensor Infrastructure*.), the system has not yet undergone clinical validation in live animals. This vital step, which involves evaluating the sensors' diagnostic performance and accuracy in detecting specific pathologies. Consequently, the potential impacts on animal welfare and the efficiency of early disease detection remain theoretical until such clinical trials are completed.

Furthermore, the scope of this implementation was restricted to a single “Living Lab” environment. Although this research farm provides a controlled setting for rigorous prototyping and further development of the digital twin, the results may not yet account for the diverse environmental and infrastructural challenges found on commercial livestock farms. Additionally, this study did not include a formal cost-benefit analysis or an evaluation of user acceptance among rural practitioners. Future research will focus on scaling these tools to decentralized “sandboxes” to assess their economic viability and to ensure that the teletriage framework aligns with the practical workflows and legal requirements of the broader veterinary profession.

## 5 Conclusion

This study demonstrates that the primary technological barriers to livestock telemedicine in the Austrian context have been effectively addressed. Our results confirm that the enterprise-grade IT infrastructure and tele-triage platforms required for remote veterinary consultation are not only available but operationally robust within a “Living Lab” environment. By successfully bridging the communication gap between the rural farm and the clinical specialist, this research establishes a proof-of-concept.

However, the transition to a fully realized model remains an ongoing process. Although the telecommunications backbone is ready for immediate deployment, the biometric sensor network currently exists as a functional prototype awaiting clinical validation in live animals. The technical feasibility of continuous data streaming has been validated, but more research is required to quantify the diagnostic accuracy and cost-effectiveness of these tools under real-word use on livestock farms.

Ultimately, this project confirms that the digital framework for modernizing rural veterinary care is no longer a theoretical ambition but a tangible technical reality. Moving forward, the successful scaling of these validated tools from the laboratory to the field will require professional engagement to modernize existing legal frameworks and the political will to support decentralized “sandboxes” for long-term socio-technical integration.

## Data Availability

The original contributions presented in the study are included in the article/supplementary material, further inquiries can be directed to the corresponding author.
