# Correction: Evidence-based telemedicine for veterinarian care of livestock

**DOI:** 10.3389/fvets.2026.1981249

**Published:** 2026-09-16

**Authors:** Frontiers Production Office

**Affiliations:** Frontiers Media SA, Lausanne, Switzerland

**Keywords:** Austria, PLF, precision livestock farming, sensors, telemedicine, veterinary care

The funder University of Veterinary Medicine Vienna was erroneously omitted from the Funding statement. The correct **Funding** statement reads:

“The author(s) declared that financial support was received for this work and/or its publication. This work was supported by the State of Lower Austria through the project HOLSTEIN (Holistic approach to the sustainable provision of livestock health in Lower Austria). Open access funding provided by University of Veterinary Medicine Vienna.”

The original version of this article has been updated.

